# The T-Top Technique for Tandem Lesions: A Single-Center Retrospective Study

**DOI:** 10.3390/jcm14092945

**Published:** 2025-04-24

**Authors:** Daniele Giuseppe Romano, Raffaele Tortora, Matteo De Simone, Giulia Frauenfelder, Alfredo Siani, Ettore Amoroso, Gianpiero Locatelli, Francesco Taglialatela, Gianmarco Flora, Francesco Diana, Renato Saponiero

**Affiliations:** 1 Unit of Interventional Neuroradiology, University Hospital Salerno, Via San Leonardo, 1, 84131 Salerno, Italy; dromano@unisa.it (D.G.R.); rtortora@unisa.it (R.T.); gianmaflor1@gmail.com (G.F.); 2 Department of Medicine, Surgery and Dentistry “Scuola Medica Salernitana”, University of Salerno, Via S. Allende, 84081 Baronissi, Italy; 3 Neuroanatomy Unit, BrainLab, Mercato San Severino, 84085 Salerno, Italy; 4 Neurosurgery Unit, University Hospital “San Giovanni di Dio e Ruggi D’Aragona”, 84131 Salerno, Italy; ettore.amoroso@sangiovannieruggi.it; 5 Unit of Neuoradiology, Department of Diagnostic Imaging, University Hospital Salerno, Via San Leonardo, 1, 84131 Salerno, Italy; giulia.frauenfelder@sangiovannieruggi.it (G.F.); alfredo.siani@sangiovannieruggi.it (A.S.); docloc.nrx@gmail.com (G.L.); francescotaglialat@hotmail.it (F.T.); renato.saponiero@sangiovannieruggi.it (R.S.); 6 Interventional Neuroradiology, Vall d’Hebron University Hospital, Passeig de la Vall d'Hebron, 119-129, 08035 Barcelona, Spain; f.diana@unilink.it; 7 Stroke Research Group, Vall d’Hebron Research Institute, 08035 Barcelona, Spain

**Keywords:** endovascular neurosurgery, tandem occlusions, tandem lesions, internal carotid artery (ICA) stenosis, middle cerebral artery (MCA) stenosis, stroke, new technique

## Abstract

**Background**: Tandem Lesions (TLs) or Tandem Occlusions (TOs) are characterized by simultaneous high-grade stenosis or occlusion of the proximal extracranial internal carotid artery and the intracranial terminal internal carotid artery or its branches. These lesions can result in stroke and pose significant challenges to endovascular treatment. This study introduces and evaluates the “T-Top technique” as an innovative approach to address TLs, assessing its safety and technical efficacy. **Methods**: Data from acute ischemic stroke (AIS) patients treated with the T-Top technique between September 2022 and September 2023 were retrospectively analyzed. The technique involves using the pusher wire of a stent retriever as a microwire to guide a monorail angioplastic balloon to the extracranial carotid stenosis, performing angioplasty simultaneously with stent retriever anchorage. Clinical outcomes, procedural data, and safety were assessed. **Results**: Successful reperfusion (mTICI > 2b) was achieved in 91% of cases, with a median groin puncture to final recanalization time of 50 min. Favorable clinical outcomes (mRS < 3) were observed in 69% of patients, with a low mortality rate of 6% after 90 days. **Conclusions**: The T-Top technique offers a rapid and reliable strategy for TL treatment, improving reperfusion rates and clinical outcomes. Further studies are warranted to validate its efficacy in larger cohorts. This technique holds promise for enhancing endovascular treatment outcomes in patients with Tandem Lesions.

## 1. Introduction

Tandem Lesions (TLs) or Tandem Occlusions (TOs) are a clinical condition defined by the concurrent presence of high-grade stenosis in the proximal extracranial internal carotid artery and thromboembolic occlusion in the intracranial internal carotid artery or its branches, most commonly in the middle cerebral artery [1]. These lesions account for approximately 15% to 30% of all large-vessel occlusion (LVO) strokes, according to clinical trial data and recent findings from the Systematic Evaluation of Patients Treated with Neurothrombectomy Devices for Acute Ischemic Stroke and Thrombectomy in Tandem Occlusions registries [1,2,3,4]. Treating Tandem Occlusions presents a significant challenge for neurointerventionalists, due to their generally poor prognosis [5]. Currently, there is no consensus on the optimal endovascular treatment for TLs. Two different types of approaches are usually performed in patients with TLs; the first is based on the immediate release, during the mechanical thrombectomy procedure, of a carotid stent in order to achieve immediate recanalization of the cervical tract of the ICA, while the second involves performing balloon angioplasty treatment, usually performed together with thromboaspiration using a large-caliber catheter, and crossing the stenotic tract of the cervical ICA, which will then be treated in the following days or weeks with various endoscopic or surgical approaches or medical therapy alone. It can be intuitively understood how both approaches have risks and benefits.

In addition, several studies suggest that the most effective approach to the management of TLs depends on the experience of the center, regarding both which stenosis to treat first and with what timing [6,7,8,9,10,11].

**Figure 1:**
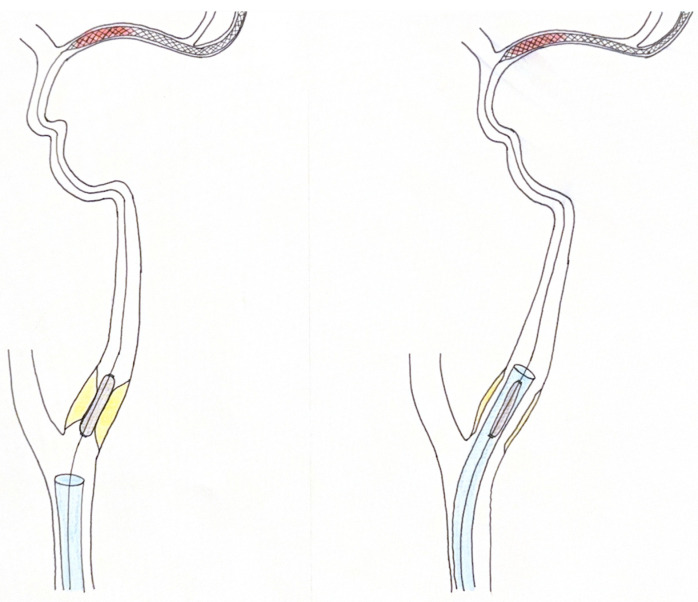
An illustrative image of the ‘’T-Top technique’’. **Left**: For the first step, after the deployment of the stent retriever at the level of intracranial occlusion, a monorail balloon, fitted over the pusher wire of the stent retriever, is employed to perform an angioplasty maneuver to resolve the extracranial carotid lesion. **Right**: During the balloon’s deflation, an 0.88” long sheath extends over the stenosis, and it is possible to perform mechanical thrombectomy and solve the intracranial occlusion.

In this article, we present our innovative approach for treating TLs, which we have named the “T-Top technique”. This technique involves using the stent retriever pusher wire (0.015–0.018″), once released at the level of the intracranial occlusion site, to guide a compatible monorail angioplastic balloon (0.018″) to the level of the extracranial carotid stenosis. Concurrently with stent retriever anchorage, angioplasty is conducted on the extracranial stenosis, effectively addressing the stenosis while utilizing the guiding catheter (Figure 1). After this step, an intermediate catheter is brought through the pusher wire, in contact with proximal medium portion of the stent retriever, to perform a combined technique for the recanalization of the intracranial closed vessel. When cranial flow has been successfully restored, a 0.014 microwire, inside the guiding catheter, is passed over the extracranial stenosis and a carotid stent is released, achieving complete extracranial recanalization and resolving the TL. The “T-Top technique” differs from traditional anterograde and retrograde approaches because the use of the stent retriever pusher wire for angioplasty guidance minimizes the procedural time. This simultaneous approach allows for the rapid restoration of intracranial flow, while reducing the risks associated with separate, i.e., two-stage treatment of intracranial and extracranial occlusions.

The aim of this single-center study was to assess the safety and technical efficacy of the “T-Top technique” to treat TLs.

## 2. Materials and Methods

### 2.1. Study Design and Clinical Assessment

In this single-center study, data from AIS patients were retrospectively analyzed after treatment with the T-Top technique due to an extracranial/intracranial TO in the period between September 2022 and September 2023. Assessment of baseline characteristics, procedural data, and the clinical outcome achieved was carried out using prospectively collected databases. All principles in the Declaration of Helsinki were followed, and the Italian laws on privacy (Art. 20–21, DL 196/2003), as published in the Official Journal, volume 190, 14 August 2004, which explicitly waives the need for ethical approval for the use of anonymous data, were respected. The Institution committee approved (ref.12/2025) the anonymized use of patient data. The inclusion criteria were as follows: evidence of a proven high-grade stenosis (≥70%, according to North American Symptomatic Carotid Endarterectomy Trial [NASCET] criteria) and acute occlusion of the extracranial ICA ipsilateral to an acute intracranial large-vessel occlusion (LVO) or medium-vessel occlusion (MEVO), based on computed tomography angiography (CTA) or digital subtraction angiography (DSA). It is known that TLs can also result from acute embolic lesions, which often have a soft thrombus through which the guiding catheter can be easily advanced; however, clinical history (e.g., atrial fibrillation) and angiographic evidence guided us safely.

All eligible patients received intravenous thrombolysis (IVT) based on the judgment of the attending neurologist, according to the guidelines of the Italian Stroke Association. The patients were included independently of the administered antiplatelet regimen. There were no general limitations on baseline variables. The angiographic results were graded locally according to the modified thrombolysis in cerebral infarction (mTICI) score. The National Institute of Health Stroke Scale (NIHSS) and modified Rankin Scale (mRS) parameters were assessed by a stroke neurologist. All values are presented as the median and interquartile range (IQR) or mean and standard deviation (SD) for continuous variables, and as the frequency and percentage for categorical variables.

### 2.2. Endovascular Technique

**Figure 2:**
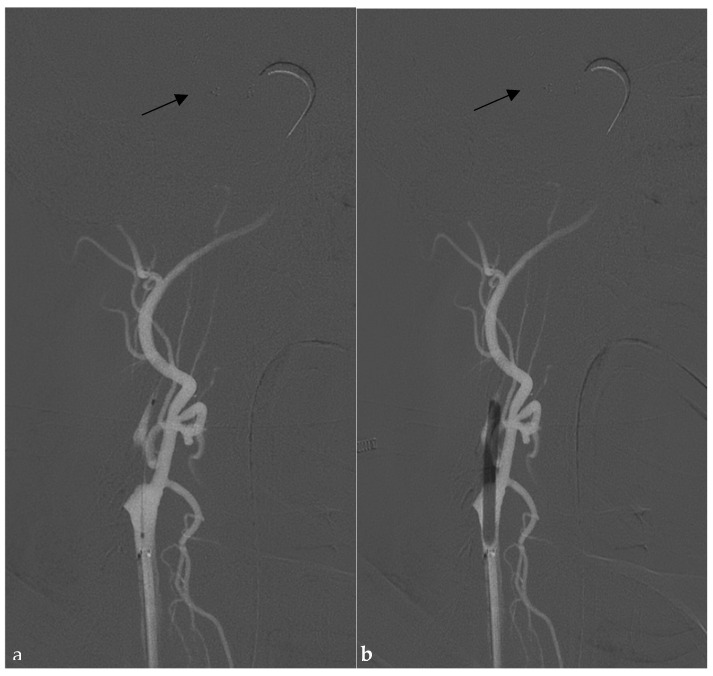
The roadmap from the CCA, 45–60° oblique projection. (**a**) A balloon angioplasty 0.018” monorail system (Sterling, Boston Scientific) is located at the level of the ICA stenosis (in this case, proximally to the origin) and (**b**) inflated. Note the control of the intracranial stent retriever’s position in the MCA during this maneuver. Black arrow: stent retriever deployment at the M1-M2 segment.

**Figure 3:**
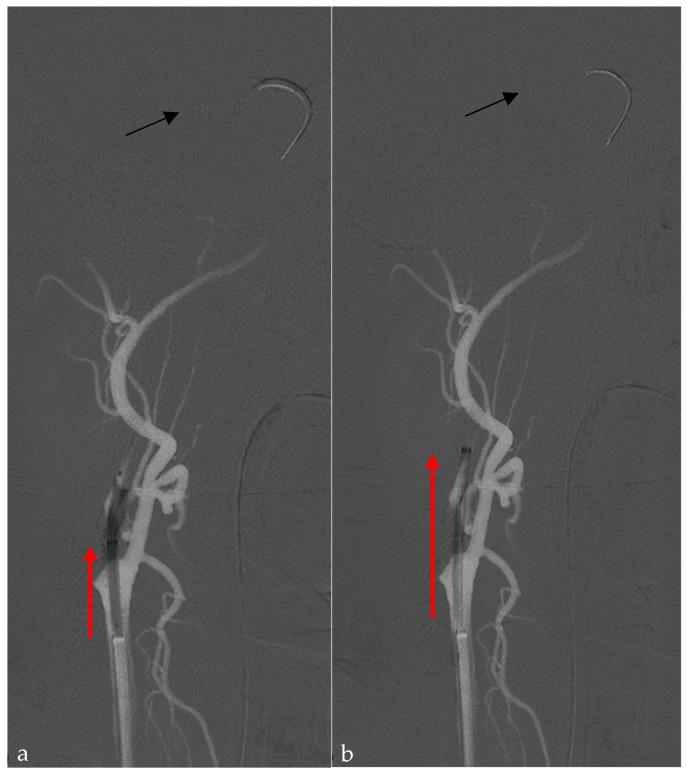
A roadmap from the CCA, 45–60°oblique projection. (**a**) During balloon deflation, the guide catheter is brought over the stenosis (red arrow). (**b**) The guide catheter in the final position after the complete deflation of the balloon catheter at the middle cervical segment of the ICA. Note the same position of the stent retriever (black arrow) after this passage.

Briefly, with the patient under general anesthesia or deep sedation, in cases of TL, an 8 French guide catheter is advanced to the distal common carotid artery (CCA). An 0.021” 160–167 cm microcatheter over a 0.014 microwire is navigated distally to the closed vessel, and a stent retriever (SR), usually 6 × 40–50 mm for LVO (large-vessel occlusion) or 3–40 mm for MEVO (medium-vessel occlusion), is deployed. After removal of the microcatheter, the A-P projection is focused on the target intracranial vessel and stent retriever, while 45–60° oblique projection is used for the depiction of the distal CCA and proximal extracranial ICA. (Sterling, Boston Scientific, Boston, MA, USA), is located at the level of the stenosis or occlusion and inflated under continuous control of the stent retriever position (Figure 2a,b), with subsequent use of the same balloon angioplasty, during its deflation, to bring the guide catheter upwards over the stenosis (Figure 3a,b).

**Figure 4:**
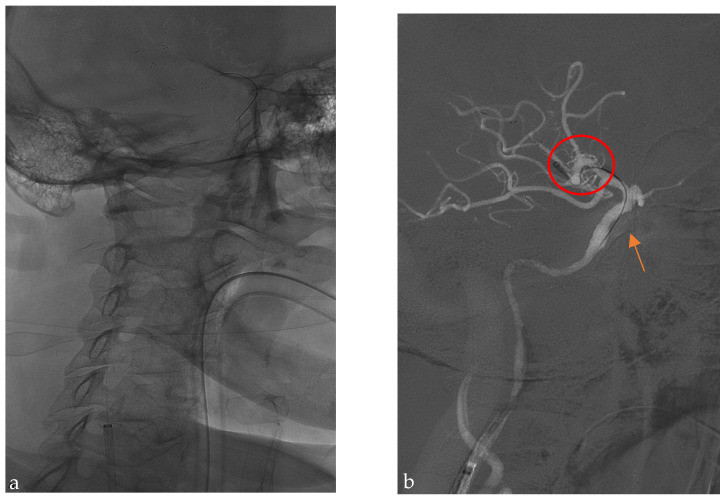
(**a**) After the ‘’T-Top technique’’, the guide catheter is stable, and it is possible to perform mechanical thrombectomy to remove the intracranial clot with the stent retriever still in position at the M1-M2 segment. (**b**) An aspiration catheter (in this case, a 0.062″ inner lumen) is engaged with the proximal third of the stent retriever; then, a combined technique is performed to perform a mechanical thrombectomy. Orange arrow: the stent retriever at M1-M2; red circle: the aspiration catheter in front of the intracranial clot.

After these steps, the stent retriever’s pusher wire is used to guide the intermediate aspiration catheter in front of the clot. The catheter’s inner diameter is chosen according to the vessel size (0.72″–0.62″ for LVO; 0.43″ for MEVO). This combined technique aims to optimize clot removal and achieve a first-pass effect efficiently (Figure 4).

**Figure 5:**
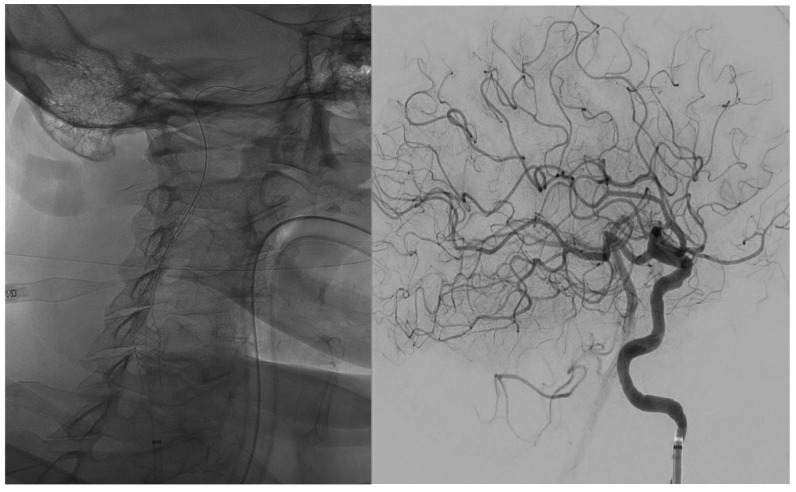
The 45–60°oblique projection. **Right**: The final result with the positioning of the carotid stent over a 0.014′ microwire, in order to completely treat the extracranial carotid lesion. **Left**: The final LL projection angiography shows a TICI 3.

When the intracranial flow has been restored, an XperCT in angiosuite is performed to determine the eventual presence of an intracranial hemorrhage (ICH) or subarachnoid hemorrhage (SAE). Then, a bolus of Cangrelor (with maintenance for 1 h) is injected intravenously prior to inserting a 0.014″ wire, used for the close air support (CAS) maneuver (Figure 5). This additional imaging step ensures the safety of the procedure by allowing the early detection of potential complications such as intracranial hemorrhage. However, it adds a few minutes to the overall procedural time, which explains why the median time from groin puncture to final revascularization is longer than the time to carotid stent deployment.

A carotid stent system is pushed to the level of the stenosis or occlusion and implanted under continuous control. After 10–15 min, it is usual to perform an angiographic check of intra–extracranial flow; at 30–40 min, a bolus of Ticagrelor (180 mg) is injected through a nasogastric tube, and after 30 min endovenous (ev.), maintenance of the cangrelor is interrupted. At 18–24 h after the procedure, a CT scan must be performed, and a dual antiplatelet therapy (DAPT) switch must be established with Ticagrelor 90 mg × 2/die plus Cardioasprin (ASS) 100 mg/die. This therapeutic protocol is used for 3 months and subsequently replaced with single antiplatelet therapy (SAPT) (ASS 100 mg) for 3 months.

## 3. Results

A total of 58 patients with acute TL were treated with the “T-Top” technique” in the period between September 2022 and September 2023. The median age was 67 (IQR 38–92), and 74% of patients were male (Table 1). Among the 58 patients, 95% (55/58) of TLs were of the anterior circulation: in the distal ICA in 29% of patients (17/58), MCA M1 in 50% (29/58), and MCA M2 in 15.5% (9/58) [12]. Only 5% of patients (3/58) had a basilar artery occlusion with simultaneous sub-occlusive stenosis of the extracranial vertebral artery. The median baseline NIHSS at admission was 14 (IQR 5–19), and the median Alberta Program Early CT Score (ASPECTS) based on initial non-contrast computed tomography was 8 (IQR 5–10). In 69% of cases (40/58), IVT was given prior the mechanical thrombectomy. The “T-Top” technique was correctly performed in all cases.

The median groin puncture to stent retriever deployment time was 23 min (IQR 12–40), and the median groin puncture to final revascularization time was 50 min (IQR 22–65). To further demonstrate the efficiency of the T-Top technique, we analyzed key procedural time metrics: (1) Angioplasty time: the median time from groin puncture to completion of the balloon angioplasty was 12 min (IQR: 9–16 min); (2) Time to first pass: the median time from groin puncture to stent retriever deployment for the first pass was 22 min (IQR: 15–30 min); (3) Time to stenting: for cases requiring stenting of the extracranial carotid artery, the median time from groin puncture to stent deployment was 38 min (IQR: 32–45 min).

These results demonstrate the ability of the T-TOP technique to achieve rapid and efficient treatment of Tandem Occlusions, underscoring its potential advantages in reducing the overall procedural time while maintaining high rates of successful reperfusion (mTICI ≥ 2b).

Overall, successful reperfusion (mTICI > 2b) was achieved in 91% of patients (53/58), with 47% (28/58) of individuals completely reperfused (mTICI 3). First-pass complete reperfusion was achieved in 20/58 (35%) cases. The mean number of stent retriever passes was 1.1 ± 0.4. In 90% of (52/58) cases, the extracranial carotid artery occlusion was treated with acute stenting (CAS) and a possible subsequent angioplasty, without the need for pre-dilatation. However, balloon angioplasty remained an integral part of the T-Top technique, not as a formal pre-dilatation step for stent deployment, but to stabilize the extracranial carotid stenosis and facilitate intracranial reperfusion.

Among the remaining cases, in 3% (2/58), pre-dilatation with angioplasty was necessary to overcome the carotid stenosis, even before the stent retriever was deployed, and in 7% (4/58), no acute carotid stenting was performed after intracranial mechanical thrombectomy: 3/4 (75%) due to increased signs of ischemic damage with a low ASPECTS [5], and 1/4 due an intraparenchymal hemorrhage after mechanical thrombectomy.

In 10% of patients (6/58), a distal embolization occurred after the mechanical thrombectomy: in 33% (2/6) of cases, this was likely attributable to the angioplasty, as embolization occurred both in the anterior and middle cerebral artery.

Subarachnoid hemorrhage occurred in 10% of all cases, and it was probably due to the use of the stent retriever (“stripping”). Only two patients developed intracranial hemorrhage, found at postoperative CT follow-ups (after carotid stent placement and, therefore, administration of antiplatelet therapy), and the prognosis in both cases was inauspicious.

The antiplatelet therapy protocol adopted to treat the extracranial carotid lesion with stenting was as follows: in patients who have not previously undergone dual antiplatelet therapy (DAPT), we usually inject an IV bolus of Cangrelor (based on the patient’s weight), followed by IV maintenance (based on the patient’s weight) for 1 h; 30–40 min after administration of the bolus of Cangrelor, we switch to a Ticagrerol loading dose (180 mg). After 18–24 h, DAPT with a normal dose of Ticagrelor (90 mg × 2 die) and ASS (100 mg/die) is introduced. This DAPT protocol lasts for 3 months, after which a carotid EchoColor–Doppler exam is performed, and if possible, SAPT is continued for 3 months.

The median ASPECT score after 24 h was 7 (serve DS), substantially in line with the onset ASPECT score. The median NIHSS after 24 h was 6 (serve DS), that is, significantly lower than the NIHSS at onset.

A favorable clinical outcome (mRS < 2) was achieved in 36/52 patients (69%). The global mortality (MRs6) rate at 3 months in our retrospective study was 9%.

## 4. Discussion

With such a broad landscape of treatment options—where no single approach has demonstrated a clear clinical advantage—it is essential to contribute insights that refine and expand the therapeutic possibilities available to our patients. Indeed, there remains a significant gray area regarding the indications for endovascular intervention and the specific types of procedures to be recommended. In this context, it is valuable to provide contributions that highlight the efficacy and safety of interventions in well-defined clinical settings.

In this article, we showcased the effectiveness and dependability of the “T-Top” technique for treating TLs. Presently, there is no standardized guideline for the best approach to treating TLs. A key area of uncertainty is the optimal timing for ICA recanalization; in fact, a one-shot approach can be followed, i.e., during the initial procedure, when the intracranial lesion is recanalized, or as a separate procedure days after the mechanical thrombectomy. [13]. Stratifying the complexity of clinical management and moving up one more level in the single-shot treatment setting, there is an ongoing debate as to whether it is preferable to employ an anterograde approach, which involves primary recanalization of the ICA followed by mechanical thrombectomy of the intracranial large-vessel occlusion, or a retrograde strategy, which addresses the intracranial occlusion first and then recanalization of the ICA. Several studies have established that there are no significant differences between the two approaches, with a slightly lower incidence of periprocedural complications with the retrograde approach [6,14,15].

A key advantage of this technique is the short recanalization time, which was 50 min in this study. This is shorter compared to other procedural times reported in the literature: 63 min by Maus et al. [15], 88 min by Behme et al., and 103/123 min by Maus et al. for antegrade and retrograde approaches, respectively [6,16]. Moreover, our technique involves placing the stent retriever and performing angioplasty on the extracranial ICA stenosis using its pusher wire, which increases the stent retriever deployment time and enhances clot removal. This combined approach, which surpasses the extracranial stenosis after PTA with an intermediate aspiration catheter, is known to improve revascularization outcomes [12].

Our study showed a notably high rate of successful reperfusion (TICI ≥ 2b), at 91%, comparable to the 96% reported for the Revised Care technique by Maus et al. [15], and higher than the 76% reported in the Pooled Analysis from the TITAN and ETIS Registries [17]. Additionally, a comprehensive review of 22 previous studies involving 790 patients with TLs revealed a 79% success rate for achieving TICI 2b or higher [18], which is still lower than our results.

Furthermore, the rate of mRs ≤ 2 after 90 days was higher (69%) than the 57% favorable outcome rate (mRs ≤ 2 after 90 days) for the stenting patients group reported by Anadani et al. [17]. In our series, we reported only two cases of intracranial parenchymal hemorrhage (3%), and both patients had been treated with antiplatelet therapy for acute stenting. This result aligns with the 2.44% reported by Francois Zhu et al. for patients treated with mechanical thrombectomy, acute carotid stenting, antiplatelet therapy, and rTPA [18].

Another significant advantage of this technique is its safety. Assessing the cerebral parenchymal condition with an XperCT scan in the angiosuite after intracranial vessel recanalization and before intravenous antiplatelet therapy administration is, in our view, essential for ensuring the procedure’s safety. This is reflected in our low rate of intracranial parenchymal hemorrhage (3%), in line with the results of Francois Zhu et al., who analyzed a total of 790 patients (2.44%) in their review study [19]. The observed 10% rate of subarachnoid hemorrhage in our series aligns with previously reported rates in the literature. The ASTER trial [20] demonstrated comparable rates of SAH between first-line direct aspiration and stent retriever techniques. Similarly, a meta-analysis by Texakalidis et al. [21] confirmed similar rates of SAH between these two approaches. This suggests that our reported SAH rate is consistent with accepted benchmarks, further supporting the safety profile of the T-Top technique. In our case series, SAH, in these cases, was likely attributable to the stent retriever (“stripping”) mechanism during mechanical thrombectomy. Although this rate is consistent with those previously reported for both stent retriever and suction techniques, as discussed above, it remains a significant procedural complication. Importantly, the patients in our study underwent careful assessment of intracranial parenchymal status with XperCT before starting intravenous antiplatelet therapy, to reduce the risks associated with DAPT. This step is critical to balance the need for effective revascularization with the risk of hemorrhagic complications.

This study has several limitations that should be acknowledged. Firstly, its retrospective design and the relatively small sample size from a single center may limit the generalizability of the findings. Additionally, the lack of standardization in medical treatments among patients introduces potential confounding variables that could affect the outcomes. Furthermore, the short-term follow-up period of 90 days may not capture long-term outcomes and complications associated with the T-Top technique. Larger, prospective studies involving multiple centers with standardized treatment protocols are needed to validate the efficacy and safety of this approach in the broader stroke population.

## 5. Conclusions

The “T-Top technique” has been demonstrated to be a safe, efficient, and dependable strategy for treating Tandem Occlusions. Its primary advantage lies in its time-saving nature, facilitating rapid reperfusion, ensuring favorable safety outcomes, and potentially surpassing existing approaches. Importantly, this technique enables the recanalization of intracranial occlusions, while concurrently addressing extracranial carotid lesions, all within a minimal timeframe.

## Figures and Tables

**Table 1:** Baseline and peri-procedural characteristics with clinical outcomes.

Age (years), median (IQR)	67 (38–92)
Male, n (%)	43 (74)
Arterial hypertension, n (%)	40 (69)
Atrial fibrillation, n (%)	13 (22)
Diabetes mellitus, n (%)	8 (14)
NIHSS baseline, median (IQR)	14 (5–19)
IVT, n (%)	40 (69)
ASPECT baseline, median (IQR)	8 (5–10)
*Intracranial occlusion site*	
ICA, n (%)	17 (29)
MCA M1, n (%)	29 (50)
MCA M2, n (%)	9 (16)
BA, n (%)	3 (5)
*Procedural data*	
General anesthesia, n (%)	40 (69)
Onset—admission [min], median (IQR)	180 (90–410)
Admission—groin puncture [min], median (IQR)	45 (20–63)
Groin puncture—SR deployment [min], median (IQR)	23 (12–40)
Groin puncture—final recanalization [min], median (IQR)	50 (22–65)
Groin puncture—balloon angioplasty [min], median (IQR)	30 (15–45)
First-pass complete reperfusion (mTICI 3), n (%)	20 (35)
First-pass successful reperfusion (mTICI > 2b), n (%)	46 (79)
Overall complete reperfusion (mTICI 3), n (%)	28 (47)
Overall successful reperfusion (mTICI > 2b), n (%)	53 (91)
Number of SR passes, mean ± SD	1.1 ± 0.4
Distal embolization, n (%)	6 (10)
Subarachnoid hemorrhage, n (%)	6 (10)
Intracranial parenchymal hemorrhage, n (%)	2 (3)
*Clinical outcome*	
ASPECT 24 h, median (IQR)	7 (4–10)
NIHSS 24 h, median (IQR)	6 (3–19)
NIHSS at discharge, median (IQR)	5 (2–12)
mRS < 2 at discharge, n (%)	35/58 (60)
mRS < 2 after 90 days, n (%)	36/52 (69)
Periprocedural mortality, n (%)	2/58 (3)
Mortality after 90 days, n (%)	3/52 (6)

## Data Availability

The data presented in this study are available on request from the corresponding author.

## Abbreviations

The following abbreviations are used in this manuscript:

TL	Tandem Lesion
mRS	Modified Rankin Scale
NIHSS	National Institutes of Health Stroke Scale
ASPECT	Alberta Stroke Program Early CT Score
IVT	Intravenous thrombolysis
SR	Stent retriever
CCA	Common carotid artery
ICA	Internal carotid artery
MCA	Middle cerebral artery
BA	Basilar artery
mTICI	Modified thrombolysis in cerebral infarction
AIS	Acute ischemic stroke
TICI	Thrombolysis in cerebral infarction
LL	Lateral lumbar (projection)
T-Top	Refers to the specific technique being discussed in the manuscript
